# Tumor Necrosis Factor-Mediated Survival of CD169^+^ Cells Promotes Immune Activation during Vesicular Stomatitis Virus Infection

**DOI:** 10.1128/JVI.01637-17

**Published:** 2018-01-17

**Authors:** Prashant V. Shinde, Haifeng C. Xu, Sathish Kumar Maney, Andreas Kloetgen, Sukumar Namineni, Yuan Zhuang, Nadine Honke, Namir Shaabani, Nicolas Bellora, Mareike Doerrenberg, Mirko Trilling, Vitaly I. Pozdeev, Nico van Rooijen, Stefanie Scheu, Klaus Pfeffer, Paul R. Crocker, Masato Tanaka, Sujitha Duggimpudi, Percy Knolle, Mathias Heikenwalder, Jürgen Ruland, Tak W. Mak, Dirk Brenner, Aleksandra A. Pandyra, Jessica I. Hoell, Arndt Borkhardt, Dieter Häussinger, Karl S. Lang, Philipp A. Lang

**Affiliations:** a Department of Molecular Medicine II, Medical Faculty, Heinrich Heine University, Düsseldorf, Germany; b Department of Gastroenterology, Hepatology, and Infectious Diseases, Heinrich-Heine-Universität Düsseldorf, Düsseldorf, Germany; c Department of Pediatric Oncology, Hematology and Clinical Immunology, Center for Child and Adolescent Health, Heinrich Heine University, Medical Faculty, Düsseldorf, Germany; d Computational Biology of Infection Research, Helmholtz Center for Infection Research, Braunschweig, Germany; e Institute of Virology, TU Munich, Munich, Germany; f Institute of Immunology, Medical Faculty, University of Duisburg-Essen, Essen, Germany; g Department of Immunology and Microbial Science, The Scripps Research Institute, La Jolla, California, USA; h Instituto Andino Patagónico de Tecnologías Biológicas y Geoambientales (IPATEC), Universidad Nacional del Comahue-CONICET, Bariloche, Argentina; i Institute for Virology of the University Hospital Essen, University of Duisburg-Essen, Essen, Germany; j Department of Cell Biology, Vrije University Medical Center, Amsterdam, Netherlands; k Institute of Medical Microbiology and Hospital Hygiene, Heinrich-Heine-Universität Düsseldorf, Düsseldorf, Germany; l Division of Cell Signalling and Immunology, School of Life Sciences, University of Dundee, Dundee, United Kingdom; m Laboratory of Immune Regulation, School of Life Science, Tokyo University of Pharmacy and Life Sciences, Tokyo, Japan; n Institute of Molecular Immunology, Technische Universität Munich and Helmholtz Zentrum Munich, Munich, Germany; o Division of Chronic Inflammation and Cancer, German Cancer Research Center (DKFZ), Heidelberg, Germany; p Institut für Klinische Chemie und Pathobiochemie, Klinikum rechts der Isar, Technische Universität Munich, Munich, Germany; q Princess Margaret Cancer Center, University Health Network, Toronto, Ontario, Canada; r Department of Infection and Immunity, Experimental and Molecular Immunology, Luxembourg Institute of Health, Esch-sur-Alzette, Luxembourg; s Odense Research Center for Anaphylaxis (ORCA), Department of Dermatology and Allergy Center, Odense University Hospital, University of Southern Denmark, Odense, Denmark; t Department of Rheumatology, Hiller Research Center Rheumatology, Heinrich-Heine-Universität Düsseldorf, Düsseldorf, Germany; u German Cancer Consortium (DKTK), partner site Munich, Germany; v German Center for Infection Research (DZIF), partner site Munich, Germany; w Center for Translational Cancer Research (TranslaTUM), Technical University of Munich, Munich, Germany; Hudson Institute of Medical Research

**Keywords:** TNF, MALT1, innate immunity, interferon, NF-κB, innate immunity, interferons, tumor necrosis factor

## Abstract

Innate immune activation is essential to mount an effective antiviral response and to prime adaptive immunity. Although a crucial role of CD169^+^ cells during vesicular stomatitis virus (VSV) infections is increasingly recognized, factors regulating CD169^+^ cells during viral infections remain unclear. Here, we show that tumor necrosis factor is produced by CD11b^+^ Ly6C^+^ Ly6G^+^ cells following infection with VSV. The absence of TNF or TNF receptor 1 (TNFR1) resulted in reduced numbers of CD169^+^ cells and in reduced type I interferon (IFN-I) production during VSV infection, with a severe disease outcome. Specifically, TNF triggered RelA translocation into the nuclei of CD169^+^ cells; this translocation was inhibited when the paracaspase MALT-1 was absent. Consequently, MALT1 deficiency resulted in reduced VSV replication, defective innate immune activation, and development of severe disease. These findings indicate that TNF mediates the maintenance of CD169^+^ cells and innate and adaptive immune activation during VSV infection.

**IMPORTANCE** Over the last decade, strategically placed CD169^+^ metallophilic macrophages in the marginal zone of the murine spleen and lymph nodes (LN) have been shown to play a very important role in host defense against viral pathogens. CD169^+^ macrophages have been shown to activate innate and adaptive immunity via “enforced virus replication,” a controlled amplification of virus particles. However, the factors regulating the CD169^+^ macrophages remain to be studied. In this paper, we show that after vesicular stomatitis virus infection, phagocytes produce tumor necrosis factor (TNF), which signals via TNFR1, and promote enforced virus replication in CD169^+^ macrophages. Consequently, lack of TNF or TNFR1 resulted in defective immune activation and VSV clearance.

## INTRODUCTION

Innate immune activation is crucial for inducing antiviral immunity through cytokine production and adaptive immune priming (1). Splenic marginal-zone macrophages and metallophilic marginal-zone macrophages play an important role in eliminating blood-borne bacteria, parasites, and viral pathogens (2, 3). Metallophilic macrophages were originally described when rat splenic marginal-zone macrophages were stained by iron and silver impregnation (4). These metallophilic macrophages express the lectin-like hemagglutinin CD169, which was identified using a monoclonal antibody, MOMA-1 (5–7). CD169^+^ macrophages are increasingly recognized to play a pivotal role in host defense (8). CD169^+^ macrophages (referred to here as CD169^+^ cells), specifically allow early viral replication to promote innate immune recognition and antigen presentation (9). The absence of CD169^+^ cells results in reduced type I interferon (IFN-I) production, reduced B-cell activation, and development of severe disease during viral infection (10, 11). B-cell-derived lymphotoxin alpha (Ltα) and lymphotoxin beta (Ltβ) drive the maintenance of CD169^+^ cells in spleen and lymph node tissue (10, 12, 13). Consequently, B-cell-deficient mice exhibit fewer CD169^+^ cells and limited immune activation, including the production of IFN-I (13, 14). However, the factors promoting survival and the presence of CD169^+^ cells after viral infection have not yet been sufficiently studied.

IFN-I triggers strong inhibitory effects on viral replication and is crucial for preventing severe infections with the vesicular stomatitis virus (VSV) model system (1, 15). This system can be used as a laboratory system for immune recognition during viral infection, as a vaccine vector system, as a tool for viral transduction, and as an oncolytic virus (16, 17). Clearance of VSV depends heavily on IFN-I and the presence of neutralizing antibodies (15, 18). VSV has been used as a murine model of viral infections to study the innate immune response and virus replication in secondary lymphoid organs and the central nervous system (CNS) (19–21). Pathology in VSV infection is seen particularly during infection of the CNS; this pathology includes paralysis and death after infection with VSV (22). Accordingly, mice deficient in IFN-α/β receptor (IFNAR) signaling exhibit paralysis and the presence of VSV in the CNS (15). Consistently, IFN-I can inhibit VSV replication in neurons, and defects in IFN-stimulated genes (ISGs) in the CNS tissue trigger pathology during VSV infection (23, 24). During infection with low doses of VSV, replication of VSV in CD169^+^ cells in the spleen and lymph node tissue is important for inducing protective immunity and preventing CNS infection (9, 10). The VSV backbone is also used during vaccination to induce protective immunity against viruses such as Ebola virus (25).

The role of tumor necrosis factor (TNF) in marginal-zone development and marginal-zone function is controversial. Although reports show that marginal-zone development is impaired and fewer marginal-zone macrophages are present in TNF-deficient and p55-TNFR (tumor necrosis factor receptor 1 [TNFR1])-deficient mice (26), other reports suggest that TNF triggers marginal-zone macrophage depletion after infection (27, 28). It has also been shown that TNFR1-deficient mice are less susceptible to West Nile virus infection as a result of an uncompromised blood-brain barrier (29). However, other studies utilizing herpes simplex virus 1 as an infection model showed that TNFR1-deficient mice are more susceptible to virus infection (30, 31). It is clear that TNF-deficient mice exhibit CD169^+^ cells in the spleen, whereas this cell population is absent in *Ltα*^−/−^ mice (26, 27). Furthermore, the production of neutralizing antibodies and the proliferation of antiviral T cells can be induced in TNF-deficient animals (28, 32). These findings suggest that TNF, which is crucial for overcoming bacterial infections (33–36), plays a minor role in antiviral immunity.

In this study, we found that absence of TNF reduced the number of CD169^+^ cells; inhibited IFN-I production; and, consequently, led to a severe disease outcome during infection with VSV. These effects were mainly transmitted by TNFR1 and were dependent on canonical nuclear factor κB (NF-κB).

## RESULTS

### TNF production by CD11b^+^ Ly6C^+^ Ly6G^+^ cells following VSV infection.

**FIG 1:**
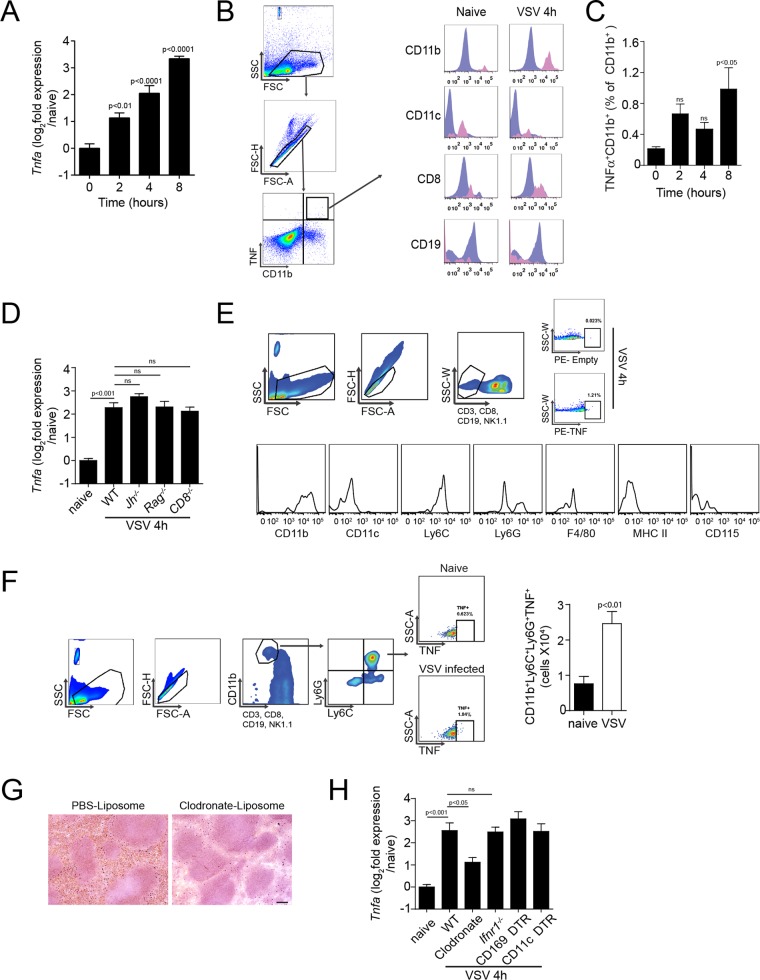
Vesicular stomatitis virus infection leads to infiltration of TNF-producing phagocytes. (A to F) WT mice were infected with 2 × 10^8^ PFU VSV. (A) TNF-α mRNA expression levels in WT spleen tissue were determined at the indicated time points after infection (*n* = 4 to 10). (B) Surface molecule expression of CD11b, CD11c, CD8, and CD19 on TNF^+^ cells 4 h after infection (purple gate, whole spleen; pink gate, TNF^+^ cells; one representative result out of 5 is shown). Numbers below the histograms indicate fluorescence intensities. FSC, forward scatter; SSC, side scatter. (C) Splenocytes from WT mice were stained for intracellular-TNF production. TNF^+^ CD11b^+^ cells were determined as percentages of total CD11b^+^ cells (*n* = 5). (D) TNF-α mRNA expression in the spleens of WT, *Jh^−/−^*, *Rag^−/−^*, and *CD8^−/−^* mice was determined 4 h after infection (*n* = 5 or 6). (E) Surface molecule expression of TNF-producing cells 4 h after infection. CD3^−^ CD8^−^ CD19^−^ NK1.1^−^ cells were further characterized for expression of CD11b, CD11c, Ly6C, Ly6G, F4/80, MHC-II, and CD115 on TNF^+^ cells (*n* = 6). The numbers in the boxes are percentages of the population positive for TNF expression. (F) CD3^−^ CD8^−^ CD19^−^ NK1.1^−^ CD11b^+^ Ly6C^+^ Ly6G^+^ TNF^+^ cells were quantified in spleen tissue 4 h after infection (*n* = 6). (G) Mice were injected with liposomes containing phosphate-buffered saline (PBS; PBS liposomes) or clodronate liposomes, and spleen tissue was harvested after 24 h. Sections of snap-frozen spleen tissue were stained with anti-F4/80 antibodies (*n* = 3). (H) TNF-α mRNA expression was determined in the spleens of WT, clodronate-treated WT, *Ifnar^−/−^*, DT-treated CD169-DTR, and CD11c-DTR mice 4 h after infection (*n* = 6). ns, not significant. The error bars indicate SEM.

TNF can be detected during an infection with VSV (32, 37). Consistently, we found that TNF expression levels were higher in the spleen after infection with VSV than in uninfected controls (Fig. 1A). Backgating of intracellular-TNF-producing cells showed that TNF-producing cells are a heterogeneous CD11b^+^ CD19^−^ population (Fig. 1B and C). Therefore, we hypothesized that TNF was likely not expressed by B or T cells during infection. Accordingly, we observed TNF mRNA expression levels in *Cd8^−/−^*, B-cell-deficient *Jh^−/−^*, and *Rag1^−/−^* mice that were comparable to those in wild-type (WT) mice (Fig. 1D). TNF-producing cells could be predominantly characterized as CD11b^+^ CD11c^−^ Ly6C^+^ Ly6G^+^ major histocompatibility complex class II negative (MHC-II^−^) (Fig. 1E). Consistent with reports that neutrophils (38, 39) and CD11b^+^ Ly6C^+^ Ly6G^+^ cells (40) are important during early defense against bacterial and viral infections via production of proinflammatory cytokines, such as interleukin 1b (IL-1b), IL-6, TNF, and IFN-I, we found a significant increase of TNF^+^ CD11b^+^ Ly6C^+^ Ly6G^+^ cells (Fig. 1F). Treatment with clodronate encapsulated in liposomes (clodronate liposomes) can deplete phagocytic cells in mice (Fig. 1G) (41, 42). Accordingly, clodronate depletion reduced TNF expression after VSV infection, suggesting a role of these phagocytic cells in the production of TNF (Fig. 1H). However, when we employed diphtheria toxin receptor (DTR)-induced specific depletion of CD169^+^ cells and CD11c^+^ cells, we did not observe a reduction in TNF production (Fig. 1H). Taken together, these findings indicate that TNF production following intravenous VSV infection is triggered by CD11b^+^ CD11c^−^ Ly6C^+^ Ly6G^+^ phagocytes.

### TNF triggers the maintenance of CD169^+^ cells during viral infection to protect animals against the development of severe disease.

**FIG 2:**
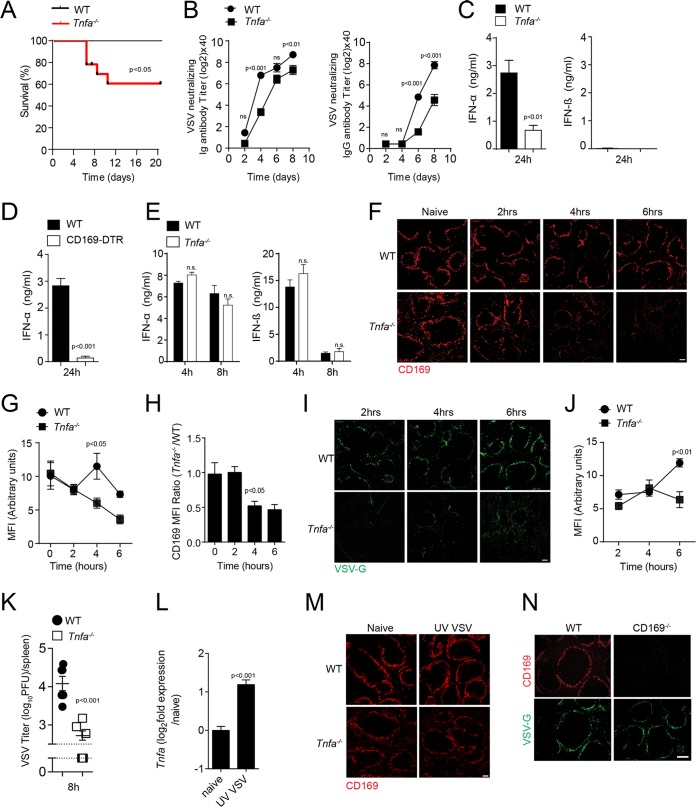
Tumor necrosis factor is required for early innate immune activation via maintenance of CD169^+^ cells during viral infection. (A to D) Mice were infected with 10^5^ PFU VSV. (A) Survival of WT and TNF-α-null (*Tnfa^−/−^*) mice was monitored for 20 days after infection (*n* = 9 to 12). (B) Titers of neutralizing total immunoglobulin (Ig) (left) and IgG (right) were determined in WT and *Tnfa^−/−^* mice at the indicated time points after infection (*n* = 7). (C) IFN-α and IFN-β concentrations were determined in the sera of WT and *Tnfa^−/−^* mice 24 h after infection (*n* = 6 to 9). (D) IFN-α levels were determined in sera from WT and CD169-DTR mice 24 h after infection (*n* = 6). (E) IFN-α and IFN-β concentrations were determined in the sera of WT and *Tnfa^−/−^* mice injected with 200 μg of poly(I·C) at the indicated time points (*n* = 3). (F) WT and *Tnfa^−/−^* mice were infected with 2 × 10^8^ PFU of VSV. Snap-frozen spleen sections were stained with anti-CD169 antibodies (clone MOMA-1) at the indicated time points (one representative result out of 6 mice is shown; scale bar = 100 μm). (G) The mean fluorescence intensity (MFI) of CD169 was quantified across spleen sections from naive and VSV-infected WT and *Tnfa^−/−^* mice using ImageJ (1 to 3 images per spleen from 3 or 4 mice were analyzed). (H) MFI from *Tnfa^−/−^* mice normalized to WT MFI. (I) Snap-frozen spleen sections from WT and *Tnfa^−/−^* mice were stained for VSV-G expression (clone Vi10) after infection with 2 × 10^8^ PFU VSV at the indicated time points (one representative result out of 6 mice is shown; scale bar = 100 μm). (J) MFI of VSV-G expression quantified across spleen sections from naive and VSV-infected WT and *Tnfa^−/−^* mice using ImageJ (1 to 3 images per spleen from 3 or 4 mice were analyzed). (K) WT and *Tnfa^−/−^* mice were infected with 10^5^ PFU VSV. Viral titers were measured in the spleens of WT and *Tnfa^−/−^* mice 8 h after infection with VSV (*n* = 6). (L) *Tnfa* mRNA expression determined in spleen tissue of WT mice before and 4 h after injection with UV-inactivated VSV (*n* = 4). (M) Spleen tissue sections were stained with anti-CD169 antibodies in WT and *Tnfa^−/−^* mice 8 h after infection with 2 × 10^8^ PFU of UV-inactivated VSV (one representative result out of 3 is shown). (N) Sections from snap-frozen spleen tissue harvested from WT and *CD169^−/−^* mice were stained for CD169 and VSV-G 7 h after infection with 2 × 10^8^ PFU VSV (*n* = 3; scale bar = 100 μm). The error bars indicate SEM.

To determine whether TNF affects the outcome after VSV infection, we infected WT and TNF-deficient mice. TNF-deficient mice developed severe VSV infection in comparison to WT mice (Fig. 2A). A neutralizing antibody titer was achieved later in TNF-deficient mice than in WT mice after infection with low doses of VSV (Fig. 2B). Since IFN-I is critical to overcome an infection with VSV (15), we measured IFN-α and IFN-β in the sera of infected animals. IFN-α production was impaired in TNF-deficient mice compared to control animals (Fig. 2C). However, IFN-β was undetectable in the sera of animals infected with 10^5^ PFU VSV (Fig. 2C). Previous findings showed that CD169^+^ cells contribute to innate immune activation in mice, not only by allowing viral replication, but also by producing IFN-I (10, 43). When we depleted CD169^+^ cells expressing diphtheria toxin receptor (CD169-DTR cells) by administering diphtheria toxin (DT) (44), we observed reduced IFN-I concentrations in the sera of infected animals (Fig. 2D). To exclude the possibility of defective innate Toll-like receptor (TLR) activation, we administered the TLR3 agonist poly(I·C). We found that the IFN-I production was intact in both WT and TNF-deficient mice (Fig. 2E). Hence, we speculated that TNF promoted the function of CD169^+^ cells and thus contributed to IFN-I production following VSV infection. Shortly after infection with VSV, the number of CD169^+^ cells in spleen tissue decreased in TNF-deficient mice compared to spleen tissue harvested from WT animals (Fig. 2F to H). To understand the reduced production of IFN-I in the absence of TNF, we monitored the virus replication in spleen tissue of WT and *Tnfa^−/−^* mice. The expression of VSV glycoprotein (VSV-G) was detected in smaller quantities in spleen tissue harvested from TNF-deficient animals than in spleen tissue harvested from WT mice after VSV infection (Fig. 2I and J). Consistently, early VSV titers after infection were lower in *Tnfa^−/−^* mice than in control mice, a condition that negatively affected antiviral immune activation (Fig. 2K). Injection of UV light (UV)-inactivated virus could increase TNF mRNA expression in WT mice (Fig. 2L). However, the decrease of CD169^+^ cells was dependent on live virus, because UV-inactivated virus did not affect CD169^+^ cells in spleen tissue of *Tnfa^−/−^* mice (Fig. 2M). These findings indicate that TNF is necessary to sustain virus replication in the early hours of infection but is dispensable for sterile innate immune activation. Notably, *CD169^−/−^* mice exhibited VSV-G expression in spleen tissue, a finding indicating that downregulation of the protein CD169 would not cause absence of virus replication (Fig. 2N). Taken together, these findings indicate that the absence of TNF results in defective antiviral innate immune activation after infection with VSV.

### CD169^+^ cell maintenance via TNFR1 results in productive VSV replication and immune activation.

**FIG 3:**
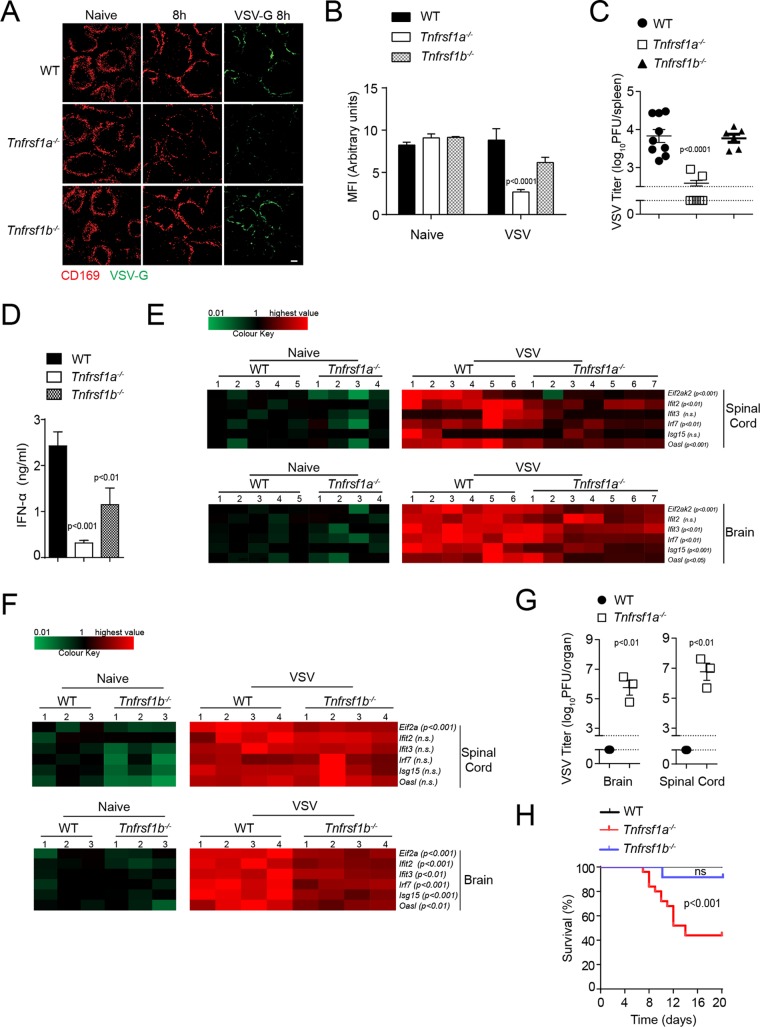
VSV replication is sustained via TNFR1 on CD169^+^ cells. (A) Spleen tissue sections from WT, *Tnfrsf1a^−/−^* (TNFR1), and *Tnfrsf1b^−/−^* (TNFR2) mice were stained with anti-CD169 and VSV-G antibodies 8 h after infection with 2 × 10^8^ PFU of VSV (one representative result out of 6 mice is shown; scale bar = 100 μm). (B) MFI of CD169 was quantified across spleen sections from WT, *Tnfrsf1a^−/−^*, and *Tnfrsf1b^−/−^* infected mice, using ImageJ (1 to 3 images per spleen from 3 or 4 mice were analyzed). (C to G) WT, *Tnfrsf1a^−/−^*, and *Tnfrsf1b^−/−^* mice were infected with 10^5^ PFU VSV. (C) Viral titers were measured in spleen tissue 8 h after infection in WT, *Tnfrsf1a^−/−^*, and *Tnfrsf1b^−/−^* mice (*n* = 6 to 9). The gap and dotted lines represent the detection limit of the virus plaque assay. (D) IFN-α concentrations were determined in the sera of WT, *Tnfrsf1a^−/−^*, and *Tnfrsf1b^−/−^* mice 24 h after infection with VSV (*n* = 6 to 9). (E) WT and *Tnfrsf1a^−/−^* mice were infected with 10^5^ PFU VSV. RNA expression levels of the indicated genes were determined in brains and spinal cords 24 h after infection (*n* = 4 to 7). The highest relative expression values (brain/spinal cord) were as follows: *Eif2ak2*, 13.72/7.98; *Ifit2*, 5.41/6.13; *Ifit3*, 35.99/34.15; *Irf7*, 68.80/54.55; Isg15, 42.54/51.23; *Oasl1*, 70.43/84.94. (F) WT and *Tnfrsf1b^−/−^* mice were infected with 10^5^ PFU VSV. RNA expression levels of the indicated genes in brains and spinal cords were determined 24 h after infection (*n* = 3 or 4). The highest relative expression values (brain/spinal cord) were as follows: *Eif2ak*, 29.84/18.21; *Ifit2*, 7.99/10.24; *Ifit3*, 41.05/51.25; *Irf7*, 166.79/88.58; Isg15, 29.78/52.99; *Oasl1*, 75.60/114.39. (G) Viral titers were measured in brain and spinal cord tissue of WT and *Tnfrsf1a^−/−^* mice once *Tnfrsf1a^−/−^* mice exhibited hind limb paralysis (*n* = *3*). (H) Survival of WT, *Tnfrsf1a^−/−^*, and *Tnfrsf1b^−/−^* mice was monitored over time after infection with VSV (*n* = 15 to 24). The error bars indicate SEM.

To further characterize the role of TNF during viral infection, we infected TNFR1- and TNFR2-deficient mice with VSV. In line with findings from TNF-deficient animals, the absence of TNFR1, but not that of TNFR2, resulted in a decrease in the number of CD169^+^ cells in spleen tissue (Fig. 3A and B). Furthermore, VSV-G production was lower in *Tnfrsf1a^−/−^* animals than in WT or *Tnfrsf1b^−/−^* mice (Fig. 3A). Consistently, VSV titers were reduced in spleen tissue shortly after infection in *Tnfrsf1a^−/−^* animals, in sharp contrast to the findings in WT and *Tnfrsf1b^−/−^* mice (Fig. 3C). Interestingly, IFN-I production was defective in *Tnfrsf1a^−/−^* mice but was also lower in *Tnfrsf1b^−/−^* animals than in WT control mice (Fig. 3D). IFN-I is necessary for the expression of antivirally active ISGs (1). Consistently, we found reduced expression of ISGs in the CNS of *Tnfrsf1a^−/−^* mice after infection with VSV (Fig. 3E). Defective ISG expression was not found to the same extent in *Tnfrsf1b^−/−^* CNS tissue (Fig. 3F). VSV can drive neuropathological symptoms by infecting the CNS (22). When we measured viral titers in the spinal cord and brain tissue of mice exhibiting hind leg paralysis, we found infectious VSV in tissue from TNFR1-deficient mice (Fig. 3G). Consequently, *Tnfrsf1a^−/−^* mice developed clinical signs of CNS infection, unlike WT and *Tnfrsf1b^−/−^* mice (Fig. 3H). Taken together, these findings suggest that TNFR1 drives antiviral defense by promoting CD169^+^ cell survival.

### TNFR1 triggers the survival of CD169^+^ cells.

**FIG 4:**
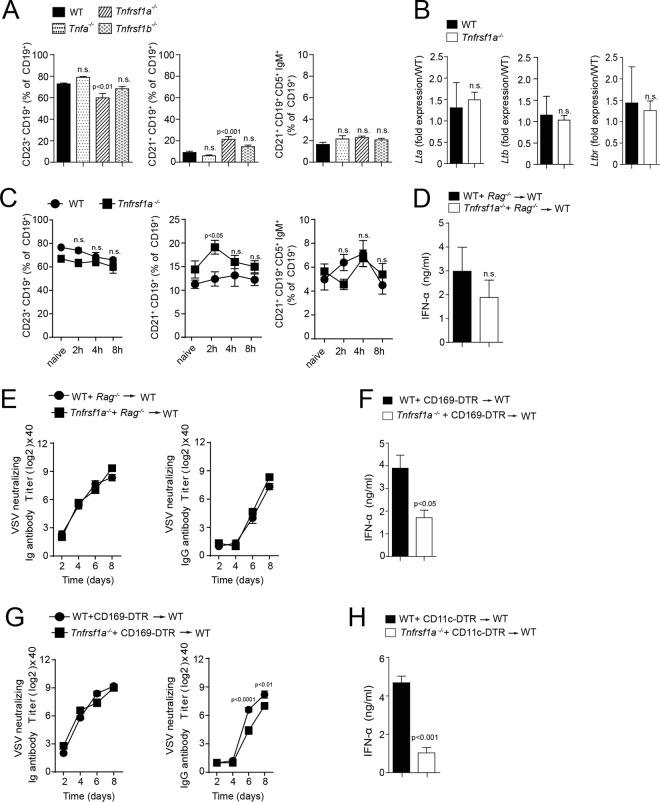
TNFR1 on CD169^+^ cells is essential for early IFN-I response. (A) Follicular B cells (CD19^+^ CD23^+^) (FB), marginal-zone B cells (CD19^+^ CD21^+^ CD23^−^) (MZB), and regulatory B cells (CD19^+^ CD21^+^ CD5^+^ IgM^+^) (RB) were analyzed in naive WT and *Tnfa^−/−^*-, *Tnfrsf1a^−/−^*-, and *Tnfrsf1b^−/−^*-deficient mice (*n* = 6). (B) Lymphotoxin α (*Ltα*), *Ltβ*, and lymphotoxin β receptor (*LtβR*) gene expression was determined in spleen tissue from WT and *Tnfrsf1a^−/−^* mice by RT-PCR (*n* = 3). (C) Splenic B-cell populations (FB, MZB, and RB) were analyzed after infection with 2 × 10^8^ PFU of VSV in WT and *Tnfrsf1a^−/−^* mice at the indicated time points (*n* = *5*). (D) IFN-α concentrations were determined 24 h after infection with 10^5^ PFU VSV in the sera of lethally irradiated mice reconstituted with either WT-*Rag^−/−^* or *Tnfrsf1a^−/−^-Rag^−/−^* bone marrow at a ratio of 1:1 (*n* = *4*). (E) Neutralizing total immunoglobulin (left) and IgG (right) antibody titers were determined in the sera of WT-*Rag^−/−^* or *Tnfrsf1a^−/−^-Rag^−/−^* reconstituted animals (*n* = *4*)**.** (F to H) Lethally irradiated WT mice were reconstituted with BM from WT or *Tnfrsf1a^−/−^* mice mixed with BM from CD169-DTR (F) and CD11c-DTR (H) mice at a 1:1 ratio. After 40 days, the mice were infected with 10^5^ PFU of VSV. Before infection, the mice were treated with 2 doses of 100 ng DT via intraperitoneal injection. (F) IFN-α concentrations were determined 24 h after infection in the sera of WT–CD169-DTR and *Tnfrsf1a^−/−^*–CD169-DTR reconstituted animals (*n* = 4 or 5). (G) Neutralizing total immunoglobulin (left) and IgG (right) antibody titers were determined in the sera of WT–CD169-DTR and *Tnfrsf1a^−/−^*–CD169-DTR reconstituted animals after infection with 10^5^ PFU VSV at the indicated time points (*n* = *4*). (H) IFN-α concentrations were determined 24 h after infection in the sera of WT–CD11c-DTR and *Tnfrsf1a^−/−^*–CD11c-DTR reconstituted mice (*n* = 4 or 5). The error bars indicate SEM.

Next, we opted to determine which factors drive the maintenance of CD169^+^ cells and enforced viral replication after viral infection. B-cell-mediated Ltβ production is important for splenic CD169^+^ cells. Hence, we wondered whether the defects in the absence of TNF were triggered by B cells. Notably, we did not observe any major changes of B-cell subsets in TNF-, TNFR1-, or TNFR2-deficient mice (Fig. 4A). Consistently, we did not see differential expression of *Ltα*, *Ltβ*, or Ltβ receptor (*LtbR*) in TNFR1-deficient mice (Fig. 4B). Additionally, we found no major differences in B-cell subsets between WT and *Tnfrsf1a^−/−^* mice after infection (Fig. 4C). Furthermore, we reconstituted lethally irradiated C57BL/6 mice with mixed bone marrow (BM) from *Rag1^−/−^* and *Tnfrsf1a^−/−^* and from *Rag1^−/−^* and WT donors at a ratio of 1:1. Mice reconstituted with *Rag1^−/−^-Tnfrsf1a^−/−^* bone marrow exhibited no significant reduction in IFN-α in the serum compared to mice reconstituted with *Rag1^−/−^*-WT bone marrow (Fig. 4D). Furthermore, there was no difference between these mice in neutralizing antibody production (Fig. 4E). To elucidate if TNFR1 deficiency specifically on CD169^+^ cells has a role in virus replication, we reconstituted lethally irradiated C57BL/6 mice with mixed bone marrow from CD169-DTR^+^ and *Tnfrsf1a^−/−^* donors, as well as CD169-DTR^+^ and WT donors, at a ratio of 1:1. We observed that the production of IFN-α was lower in the mice reconstituted with CD169-DTR^+^ and *Tnfrsf1a^−/−^* bone marrow compared to control mice reconstituted with CD169-DTR^+^ and WT bone marrow after infection with VSV and DT treatment (Fig. 4F). Furthermore, we found slightly delayed presence of VSV neutralizing antibody titers in CD169-DTR^+^–*Tnfrsf1a^−/−^* recipients compared to corresponding CD169-DTR^+^–WT recipients (Fig. 4G). CD169^+^ cells can be depleted in CD11c-DTR mice, because CD169^+^ cells exhibit intermediate expression of CD11c (10, 45). Consistently, lethally irradiated mice reconstituted with mixed bone marrow from CD11c-DTR^+^ and *Tnfrsf1a^−/−^* mice exhibited reduced concentrations of IFN-α after VSV infection compared to CD11c-DTR^+^–WT bone marrow recipients (Fig. 4H). These findings suggest that TNFR1 triggers cell-intrinsic effects on CD169^+^ cells.

**FIG 5:**
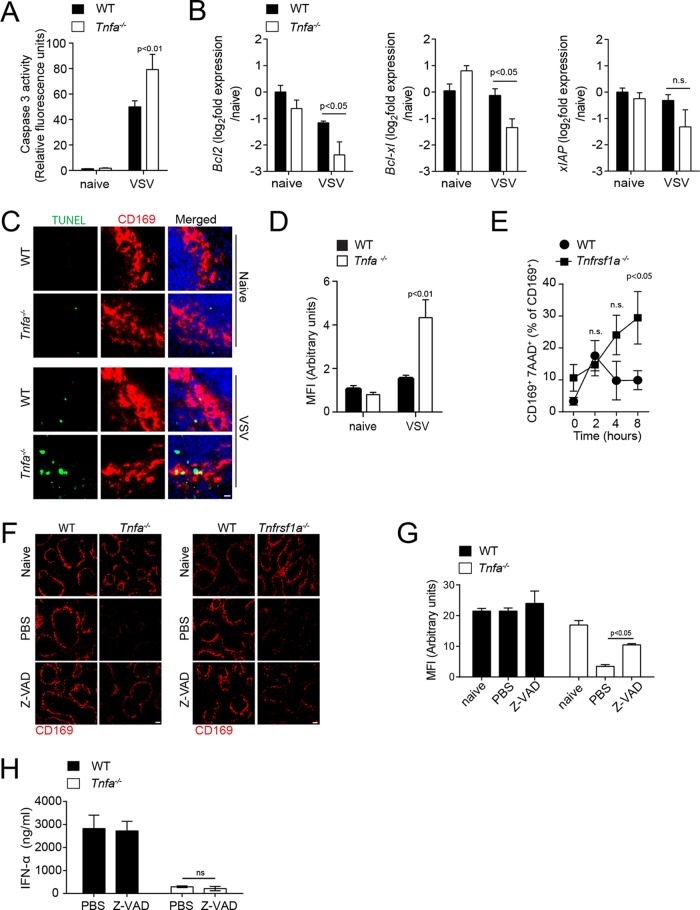
Tumor necrosis factor mediates survival of CD169^+^ cells via TNFR1. (A to E) Mice were infected with 2 × 10^8^ PFU VSV. (A) Caspase 3 activity was determined in spleen tissue harvested from WT and *Tnfa^−/−^* mice 6 h after infection with 2 × 10^8^ PFU VSV (*n* = 4 to 7). (B) *Bcl2*, *Bclxl*, and *Xiap* RNA expression was determined in spleen tissue from WT and *Tnfrsf1a^−/−^* mice 8 h after infection (*n* = 3). (C) Tissue sections from WT and *Tnfa^−/−^* mice were TUNEL stained 5 h after infection (one result representative of 3 or 4 mice is shown; scale bar = 10 μm). (D) MFI of TUNEL quantified across spleen sections from naive and VSV-infected WT and *Tnfa^−/−^* mice using ImageJ (1 or 2 images per spleen from 3 or 4 mice were analyzed). (E) At the indicated time points, the proportions of 7AAD^+^ cells among CD11b^+^ CD169^+^ cells were determined (*n* = *5*) in WT and *Tnfrsf1a^−/−^* mice. (F) WT, *Tnfa^−/−^*, and *Tnfrsf1a^−/−^* mice were treated with Z-VAD and infected with 2 × 10^8^ PFU VSV. Spleen tissue sections were stained with anti-CD169 antibodies 8 h after infection (one result representative of 3 or 4 mice is shown; scale bar = 100 μm). (G) MFI of CD169 quantified across spleen sections from naive and VSV-infected WT and *Tnfa^−/−^* mice treated with Z-VAD, using ImageJ (1 to 3 images per spleen from 3 or 4 mice were analyzed). (H) IFN-α concentrations determined 24 h after infection in the sera of Z-VAD-treated WT and *Tnfa^−/−^* mice after infection with 10^5^ PFU of VSV (*n* = 3). The error bars indicate SEM.

We speculated that TNF delivers an important survival signal for CD169^+^ cells. To determine if TNF is involved in protection against VSV-induced apoptosis, we measured caspase 3 activity on whole spleen tissue lysates. After VSV infection, caspase 3 activity was significantly higher in *Tnfa^−/−^* mice than in control animals (Fig. 5A). VSV is known to induce apoptosis and inactivates Mcl-1 and Bcl-Xl (46). To elucidate if TNF plays a role in promoting expression of antiapoptotic genes, we measured mRNA expression of *Bcl2*, *Bcl-Xl*, and *xIAP* in spleen tissue of mice after VSV infection (Fig. 5B). After VSV infection, *Bcl2* and *Bcl-Xl* expression was significantly reduced in *Tnfa^−/−^* mice compared to WT mice (Fig. 5B). To enumerate the mechanism that reduces CD169^+^ cells in TNF-deficient mice after infection, we made use of terminal deoxynucleotidyltransferase-mediated dUTP-biotin nick end labeling (TUNEL) assays. The number, as well as the mean fluorescence intensity, of TUNEL-positive CD169^+^ cells was higher in spleen tissue from TNF-deficient mice than in tissue from corresponding WT control mice (Fig. 5C and D). The proportion of CD169^+^ cells that stained positive for 7-aminoactinomycin D (7-AAD) was higher in TNFR1-deficient mice than in WT control mice 8 h after infection (Fig. 5E). Next, we wondered if we could rescue the CD169^+^ cells by injecting the pancaspase inhibitor Z-Val-Ala-Asp-fluoromethylketone (Z-VAD). Z-VAD treatment restored the presence of CD169^+^ cells in TNF-deficient animals, a finding indicating that CD169^+^ cells depend on TNF-mediated survival (Fig. 5F and G). Although treatment of TNF-deficient mice with Z-VAD-rescued CD169^+^ cells, it failed to rescue the IFN-I response, suggesting the role of TNF signaling is essential not only to prevent apoptosis, but also for IFN-I production (Fig. 5H). In summary, these findings indicate that TNF delivers a survival signal that is important for the maintenance of CD169^+^ cells in the spleen after viral infection and for IFN-I production.

### The NF-κB regulator MALT1 promotes canonical NF-κB expression, VSV replication in CD169^+^ cells, and immune activation during viral infection.

**FIG 6:**
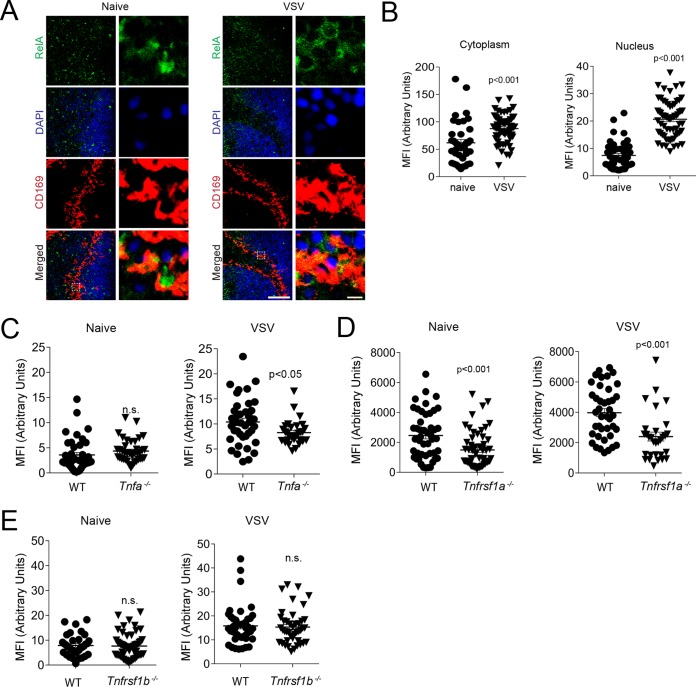
VSV infection leads to TNFR1-dependent canonical NF-κB activation in splenic CD169^+^ cells. (A to D) Sections of snap-frozen spleen tissue were harvested 4 h after infection with 2 × 10^8^ PFU VSV. (A) The sections were stained for RelA before and after infection (one representative result out of 3 is shown; scale bar = 100 μm). Enlarged images of the boxed areas in the merged images on the left side are shown on the right (scale bar = 5 μm). (B) MFI of cytoplasmic and respective nuclear RelA quantified in CD169^+^ cells from WT mice before and after VSV infection to evaluate nuclear translocation of RelA (*n* = 48 to 63). (C to E) Spleen sections from WT and *Tnfa^−/−^* (C), *Tnfrsf1a^−/−^* (D), and *Tnfrsf1b^−/−^* (E) mice were stained with anti-RelA antibodies 4 h after infection with 2 × 10^8^ PFU VSV. The MFI of RelA in the nuclei of CD169^+^ cells was determined with ImageJ software (*n* = 35 to 57). The error bars indicate SEM.

**FIG 7:**
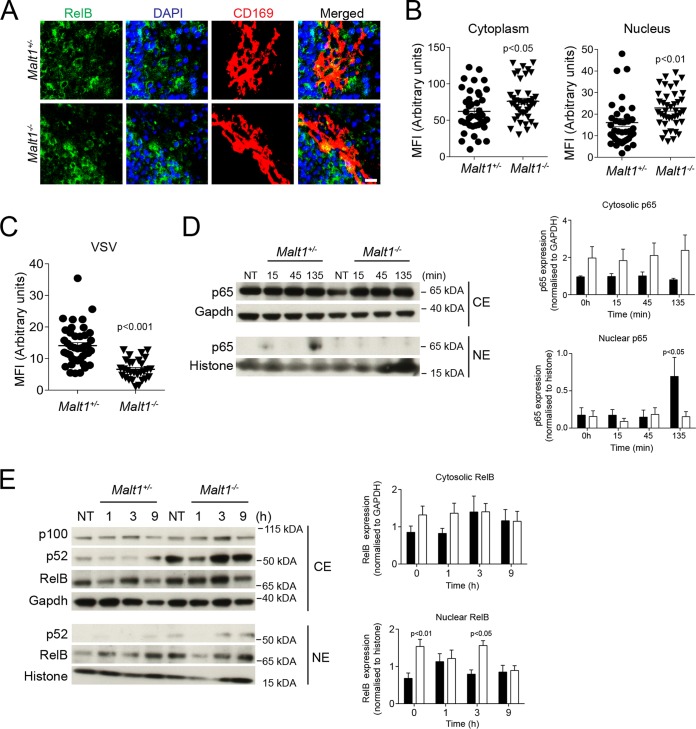
MALT1 regulates nuclear RelA expression after infection with vesicular stomatitis virus. (A) Sections from snap-frozen spleen tissue harvested from naive *Malt1^+/−^* and *Malt1^−/−^* mice were stained with anti-RelB antibodies (one representative result out of 3 is shown; scale bar = 10 μm). (B) MFI of cytoplasmic and nuclear RelB was quantified in CD169^+^ cells using ImageJ (*n* = 39 to 42). (C) Sections of snap-frozen spleen tissue from *Malt1^+/−^* and *Malt1^−/−^* mice were stained with anti-RelA antibodies 4 h after infection with 2 × 10^8^ PFU VSV. The MFI in the nuclei of CD169^+^ cells was quantified (*n* = 29 to 41). (D and E) *Malt1^+/−^* and *Malt1^−/−^* MEFs were stimulated with 100 ng/ml recombinant mouse tumor necrosis factor (rmTNF) at the indicated time points. Cytosolic extracts (CE) and nuclear extracts (NE) were harvested and probed for p65. Densitometry analysis of p65 and RelB was performed on the Western blot (WB) images from cytosolic and nuclear fractions at the indicated time points. Proteins were normalized to GAPDH or histone (*n* = 4). The error bars indicate SEM.

**FIG 8:**
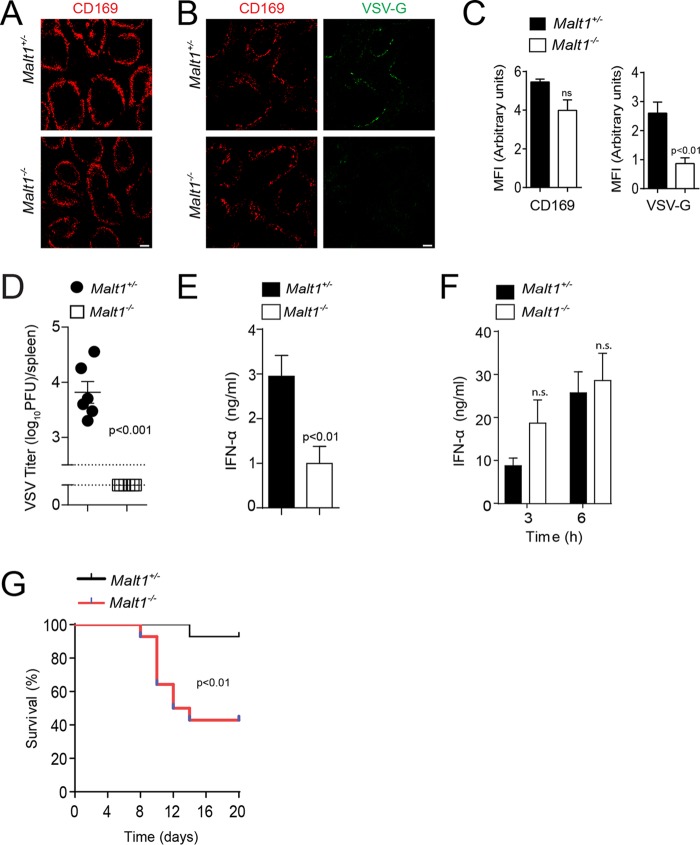
MALT1 promotes vesicular stomatitis virus replication in CD169^+^ cells and immune activation during viral infection. (A) Spleen sections from naive *Malt1^+/−^* and *Malt1^−/−^* mice were stained with anti-CD169 (one representative result out of 3 is shown; scale bar = 100 μm). (B) Sections of snap-frozen spleen tissue from *Malt1^+/−^* and *Malt1^−/−^* mice were analyzed 8 h after infection with 2 × 10^8^ PFU VSV and stained with anti-CD169 and anti-VSV-G) (one representative result out of 3 is shown; scale bar = 100 μm). (C) MFI of CD169 and VSV-G was quantified across spleen sections from VSV-infected *Malt1^+/−^* and *Malt1^−/−^* mice using ImageJ (*n* = 4). (D and E) Mice were infected with 10^5^ PFU VSV. (D) Viral titers were measured in spleen tissue of *Malt1^+/−^* and *Malt1^−/−^* mice 8 h after infection (*n* = *6*). (E) IFN-α concentrations were determined in the sera of *Malt1^+/−^* and *Malt1^−/−^* mice 24 h after infection (*n* = *6*). (F) IFN-α concentrations were determined in the sera of *Malt1^+/−^* and *Malt1^−/−^* mice injected with 200 μg poly(I·C) at the indicated time points (*n* = 3 or 4). (G) Survival of *Malt1^+/−^* and *Malt1^−/−^* animals was monitored for 20 days after infection (*n* = 13 to 14). The error bars indicate SEM.

TNF can induce NF-κB activation via TNFR1 and can promote the expression of genes driving survival and of proinflammatory cytokines (47). Furthermore, TNF is known to promote IFN-I production (48). Consistently, RelA expression was increased in the marginal zone of spleen tissue after VSV infection (Fig. 6A). We quantified cytoplasmic and nuclear expression of RelA in CD169^+^ cells. The nuclear presence of RelA in CD169^+^ cells was higher in VSV-infected mice than in naive controls (Fig. 6B). We wondered whether nuclear RelA protein expression was dependent on TNF. As expected, compared with WT control mice, VSV-infected mice exhibited reduced expression of RelA in the nuclear compartments of CD169^+^ cells in the absence of TNF (Fig. 6C). Notably, the presence of RelA was reduced in TNFR1-deficient mice, but we observed no difference in RelA expression between TNFR2-deficient mice and corresponding control mice (Fig. 6D and E). It has been reported that one of the major regulators of RelA signaling is RelB, which acts through sequestration of RelA in the cytoplasm and competitive binding of DNA (49). It has also been reported that the paracaspase MALT1 can promote canonical NF-κB signaling by cleaving RelB (50, 51). Hence, we stained spleen sections of *Malt1^+/−^* and *Malt1^−/−^* mice for RelB. Ablation of MALT1 resulted in increased levels of RelB in CD169^+^ cells in the marginal zone of the spleen (Fig. 7A and B). In turn, nuclear RelA levels were lower in CD169^+^ cells in *Malt1^−/−^* spleen tissue than in control tissue (Fig. 7C). Consistently, mouse embryonic fibroblasts (MEFs) derived from *Malt1^−/−^* mice showed reduced translocation of p65 into the nucleus after stimulation with TNF but higher expression of RelB in the nucleus (Fig. 7D and E). These findings indicate that MALT1 destabilizes RelB in marginal-zone macrophages to promote canonical NF-κB signaling. The presence of CD169^+^ cells in spleen tissue was not affected by *Malt1* before or after infection with VSV (Fig. 8A). However, the expression of VSV-G was lower in *Malt1^−/−^* mice than in control mice (Fig. 8B and C). Consistently, the numbers of infectious VSV particles were lower in spleen tissue harvested from *Malt1^−/−^* mice than in spleen tissue from control mice (Fig. 8D). Hence, serum IFN-I concentrations after VSV infection were lower in MALT1-deficient mice than in control mice (Fig. 8E). A previous report suggested that MALT1 is not required for RIG-I activation (52). Consistently, when we injected poly(I·C) into *Malt1^+/−^* and *Malt1^−/−^* mice, we found similar serum IFN-I levels in both groups (Fig. 8F). Hence, we concluded that defective IFN-I production during VSV infection was caused by reduced VSV replication early during infection. Consequently, MALT1-deficient mice succumbed to the infection, in sharp contrast to control animals (Fig. 8G).

Taken together, these findings indicate that absence of MALT1 results in reduced canonical NF-κB signaling in response to VSV infection. *Malt1*-deficient mice exhibit reduced VSV replication and immune activation.

## DISCUSSION

In this study, we found that TNF plays a crucial role in the maintenance of CD169^+^ cells early after infection with VSV. Consequently, TNF-, TNFR1-, and MALT1-deficient animals exhibited reduced immune activation and limited IFN-I production, which consequently led to severe VSV infection.

The role of TNF during viral infection is controversial and not sufficiently understood. Although reports describe activating polymorphisms in TNF, which are associated with the establishment of a chronic viral infection (53), other reports state that the same mutations are protective against chronic hepatitis B virus (HBV) infection (54). *In vitro*, TNF can propagate the replication of hepatitis C virus (HCV) (55), although HCV increases the incidence of TNF-induced apoptosis (56). On the other hand, TNF strongly inhibits influenza virus replication in porcine lung epithelial cells (57). Consistently, the attenuation of TNF signaling in a murine T-cell-independent model of HBV infection results in viral persistence (58). In turn, the application of second mitochondrion-derived activator of caspases (SMAC) mimetics enhances TNF signaling and is associated with increased clearance of HBV in this model system (59). During infection with VSV, the production of neutralizing antibodies is not defective in the absence of TNFR1 (32). Moreover, TNF can induce T-cell dysfunction and therefore promote chronic viral infection (60). Our finding that TNF is crucial for the maintenance of CD169^+^ cells in spleen tissue may be important for infections with lower doses of virus, because allowing viral replication in CD169^+^ cells is particularly important for protective adaptive immunity (9, 13). This may be crucial for the maintenance of CD169^+^ cells in spleen tissue during vaccination with attenuated virus strains or VSV vector-based vaccines (25). These findings may not be specific only for splenic CD169^+^ cells, since intranasal infection with recombinant TNF overexpressing rabies virus (RV) reduced the RV load and mortality (61).

Viral replication in CD169^+^ cells, which is promoted by TNF, contributes to improved antigen presentation. CD169^+^ cells in the marginal zone are in close contact with pathogens and are ideally situated to induce an immune response (62). Furthermore, CD169^+^ cells have been shown not only to present antigens to B cells in the lymph nodes, but also to prime T cells (11, 63). Moreover, CD169^+^ cells are important for virus-mediated IFN-I production, which prevents severe CNS infection in mice (64). Our findings show that TNF promotes maintenance of CD169^+^ cells and IFN-I production following VSV infection. Furthermore, our findings show that the translocation of RelA to the nuclei of CD169^+^ cells after VSV infection is dependent on TNF. It has been postulated that canonical NF-κB can contribute to the production of IFN-α (65, 66). However, RelA-deficient and p50-deficient MEFs can produce IFN-α after viral infection, whereas only early IFN-I transcription is reduced (67, 68). Furthermore, RelA-deficient plasmacytoid dendritic cells (pDCs) exhibited reduced IFN production after exposure to Sendai virus (69). Our findings indicate that canonical NF-κB activation can also promote early viral replication and consequently contribute to the production of IFN-I. Consistently, noncanonical NF-κB, which can inhibit canonical NF-κB signaling, is a potent inhibitor of IFN-I production (70). Hence, the paracaspase MALT1, which can cleave RelB and consequently promote canonical NF-κB signaling (50, 51), is necessary for the sufficient propagation of VSV replication and IFN-I production.

Taken together, we have found that TNF-TNFR1 signaling is crucial for protecting CD169^+^ cells and their function in innate immune activation during VSV infection.

## MATERIALS AND METHODS

### Mice, viruses, and virus titration.

*Tnfa^−/−^*, *Tnfrsf1b^−/−^*, *CD8*^−/−^, and *Rag1*^−/−^ mice were purchased from Jackson Laboratories (United States). *Tnfrsf1a^−/−^* mice have been previously described (34). *Malt1^−/−^*, *CD169^−/−^*, CD169-DTR, and CD11c-DTR mice have also been previously described (71–74). All the mice were maintained on a C57BL/6 genetic background. VSV Indiana serotype (Mudd-Summers strain) was originally obtained from D. Kolakofsky (University of Geneva, Geneva, Switzerland). VSV was propagated and titrated as previously described (13). Mice were infected with VSV via tail vein injection. In survival experiments, mice exhibiting symptoms of hind leg paralysis were considered “severe,” taken out of the experiment, and counted as dead. Blood was collected at the indicated time points after infection. VSV neutralizing antibody titers were measured by plaque reduction neutralization test (PRNT) as previously described (9, 13). Briefly, serum samples were diluted 1:40 and incubated at 56°C for 30 min. To evaluate immunoglobulin G (IgG), the serum was pretreated with 0.1 M β-mercaptoethanol. Serial 2-fold dilutions were performed for 12 steps, and the serum was incubated with 5,000 PFU of VSV. The virus and serum mixture was incubated on a Vero cell monolayer. The plates were stained with crystal violet after 24 h. To inhibit caspase activity *in vivo*, we administered three doses (2 μg/g of body weight each) of Z-VAD (Abcam, Cambridge, United Kingdom) (75, 76). For chimera experiments, mice were lethally irradiated with 10.2 Gy. After 24 h, mixed bone marrow from WT or *Tnfrsf1a^−/−^* and CD169-DTR, CD11c-DTR, and *Rag1*^−/−^ mice was transplanted into the irradiated mice as indicated. All mice were maintained under specific-pathogen-free conditions under the authorization of the Landesamt für Natur, Umwelt und Verbraucherschutz of North Rhine-Westphalia (LANUV NRW) in accordance with the German laws for animal protection.

### Depletion of cells.

To deplete macrophages, 200 μl clodronate liposomes was injected intravenously, and 24 h later, the mice were infected with VSV. Liposomes containing phosphate-buffered saline (PBS; PBS liposomes) were used as a control. Clodronate and PBS liposomes were provided by Nico van Rooijen (Vrije University Medical Center, Netherlands) and used as previously described (41, 42). CD169- and CD11c-expressing cells in CD169-DTR and CD11c-DTR mice were depleted by injecting 2 doses of 100 ng DT (Sigma) 1 day before and on the day of infection.

### Histology and ELISA.

Histological analysis of snap-frozen tissue was performed as previously described (9). Briefly, snap-frozen tissue sections were cut at 7-μm thickness, air dried, and fixed with acetone for 10 min. The sections were blocked with 2% fetal calf serum in phosphate-buffered saline (PBS) for 1 h and stained with anti-CD169 (final concentration, 4 μg/ml; Acris, Germany; clone MOMA-1), anti-VSV-G (final concentration, 1 μg/ml; produced in house; clone Vi10), anti-RelA (final concentration, 1 μg/ml; Santa Cruz Biotechnology, USA), anti-F4/80 (final concentration, 2 μg/ml; eBioscience; clone BM8), and anti-RelB (final concentration, 1 μg/ml; Cell Signaling, USA; polyclonal) for 1 h. Then, the sections were washed with PBS containing 0.05% Tween 20 (Sigma). The secondary antibodies, phycoerythrin (PE) streptavidin (final concentration, 1 μg/ml; eBioscience), anti-rabbit fluorescein isothiocyanate (FITC) (final concentration, 1 μg/ml; Thermo Fisher), and anti-goat FITC (final concentration, 1 μg/ml; Santa Cruz Biotechnology, USA), were incubated for 1 h. Then, the sections were washed with PBS containing 0.05% Tween 20 (Sigma) and mounted using fluorescence mounting medium (Dako). A caspase 3 activity assay was performed with a fluorescence assay (Cell Signaling) according to the manufacturer's instructions. TUNEL staining was performed on formalin-fixed spleen sections according to the manufacturer's instructions (Thermo Scientific, USA). Images were obtained with an LSM510 confocal microscope and an Axio Observer Z1 fluorescence microscope (Zeiss, Germany). Analysis of the fluorescence images was performed with ImageJ software. IFN-α and IFN-β (PBL Biosciences, New Jersey, USA) concentrations were determined using an enzyme-linked immunosorbent assay (ELISA) according to the manufacturers' instructions.

### Reverse transcription (RT)-PCR analyses.

RNA purification (Qiagen RNeasy kit or TRIzol) was performed according to the manufacturer's instructions. Gene expression of *Bcl2*, *Bcl-xl*, *Xiap*, *Lta*, *Ltb*, *Ifit1*, *Ifit2*, *Ifit3*, *Irf7*, *Isg15*, *Oasl1*, and *Tnfa* was performed using 6-carboxyfluorescein (FAM) and VIC probes (Applied Biosystems) and an iTAQ one-step PCR kit (Bio-Rad). For analysis, the expression levels of all the target genes were normalized to β-actin/GAPDH (glyceraldehyde-3-phosphate dehydrogenase) expression (Δ*C_T_*). Gene expression values were then calculated based on the ΔΔ*C_T_* method, using naive WT mice as a control to which all other samples were compared. Relative quantities (RQ) were determined using the following equation: RQ = 2^−ΔΔ*CT*^.

### Immunoblotting.

*Malt1^+/−^* and *Malt1^−/−^* MEFs were obtained from Jürgen Ruland (Technische Universität Munich, Munich, Germany). *Malt1^+/−^* and *Malt1^−/−^* MEFs were stimulated with 100 ng/ml murine soluble TNF (mTNF) (R&D Systems). Cytoplasmic and nuclear extracts were prepared using a nuclear protein extraction kit according to the manufacturer's instructions (Active Motif, Belgium). Immunoblots were probed with primary anti-p65 (Santa Cruz Biotechnology), anti-RelB (Cell Signaling), and anti-p100/p52 (Cell Signaling).

### Flow cytometry.

For intracellular-cytokine staining, single-cell suspended splenocytes were incubated with brefeldin A (eBioscience), followed by an additional 5 h of incubation at 37°C. After surface staining with anti-CD3, anti-CD8, anti-CD11b, anti-CD11c, anti-CD19, anti-CD115, anti-F4/80, anti-Ly6C, anti-Ly6G, anti-MHC-II, and anti-NK1.1 antibodies (all from eBioscience), the cells were fixed with 2% formalin, permeabilized with 0.1% saponin, and stained with anti-TNF antibodies (eBioscience) for 30 min at 4°C. B-cell subsets were detected in single-cell suspensions of splenocytes with anti-CD5, anti-CD19, anti-CD21, anti-CD23, and anti-IgM antibodies (all from eBioscience). BD Calibrite (BD Biosciences, USA) beads were added to the samples before acquisition with a BD LSRFortessa.

### Statistical analyses.

Data are represented with standard errors of the mean (SEM). Statistically significant differences between two groups were determined with Student's *t* test. Statistically significant differences between several groups were determined by one-way analysis of variance (ANOVA) with additional Bonferroni or Dunnett *post hoc* tests. Statistically significant differences between groups in experiments involving more than one time point were determined with two-way ANOVA (repeated measurements).
